# Cgmquantify: Python and R Software Packages for Comprehensive Analysis of Interstitial Glucose and Glycemic Variability from Continuous Glucose Monitor Data

**DOI:** 10.1109/OJEMB.2021.3105816

**Published:** 2021-08-18

**Authors:** Brinnae Bent, Maria Henriquez, Jessilyn P. Dunn

**Affiliations:** Department of Biomedical Engineering, Duke University 3065 Durham NC 27705 USA; Department of Statistical Science, Duke University 3065 Durham NC 27705 USA

**Keywords:** Continuous glucose monitor, diabetes, digital biomarkers, glycemic variability, open-source software

## Abstract

*Goal:* Continuous glucose monitoring (CGM) is commonly used in Type 1 diabetes management by clinicians and patients and in diabetes research to understand how factors of longitudinal glucose and glucose variability relate to disease onset and severity and the efficacy of interventions. CGM data presents unique bioinformatic challenges because the data is longitudinal, temporal, and there are infinite ways to summarize and use this data. There are over 25 metrics of glucose variability used clinically and in research, metrics are not standardized, and little validation exists across studies. The primary goal of this work is to present a software resource for systematic, reproducible, and comprehensive analysis of interstitial glucose and glycemic variability from continuous glucose monitor data. *Methods:* Comprehensive literature review informed the clinically-validated functions developed in this work. Software packages were developed and open-sourced through the Python Package Index (PyPi) and the Comprehensive R Archive Network (CRAN). cgmquantify is integrated into the Digital Biomarker Discovery Pipeline and MD2K Cerebral Cortex. *Results:* Here we present an open-source software toolbox called cgmquantify, which contains 25 functions calculating 28 clinically validated metrics of glucose and glycemic variability, as well as tools for visualizing longitudinal CGM data. Detailed documentation facilitates modification of existing code by the community for customization of input data and visualizations. *Conclusions:* We have built systematic functions and documentation of metrics and visualizations into a software resource available in both the Python and R languages. This resource will enable digital biomarker development using continuous glucose monitors.

## I. Introduction

Continuous glucose monitoring (CGM) systems provide real-time, dynamic glucose information by tracking interstitial glucose values throughout the day. CGMs are commonly used in Type 1 diabetes (T1D) management, with 1.2 million diabetic patients using a CGM [1], [2]. These devices have been used extensively by the T1D community, including in the Open Artificial Pancreas System Project (OpenAPS) [3], a project developed to create a patient-implemented closed loop system between a CGM and an insulin pump.

CGM data is commonly provided from CGM manufacturers as either raw glucose values (in a .csv format) or in summary reports that utilize proprietary methods to plot and summarize glucose statistics. Because these algorithms are proprietary, they cannot be externally validated by clinical researchers [4]. Additionally, the provided glucose summaries are limited and do not usually contain any information about an important clinical metric, glycemic variability.

Glycemic variability, also known as glucose variability, is an established risk factor for hypoglycemia [5] and has been shown to be a risk factor in other diabetes complications [6]. Glucose variability is mentioned in over 26000 publications indexed in PubMed at the time of this publication and is a key metric in clinical research [7]. Over 25 metrics of glucose variability have been used in the literature, which makes it difficult to examine and compare results across numerous research studies analyzing and drawing conclusions about glucose variability.

There is a need for an open-source software toolbox containing algorithms that are utilized and validated in clinical research studies. This would enable standardized glucose variability metrics and the ability to compare findings from studies that utilize different metrics of glucose variability. Its availability in an open-source programming language with a low barrier to entry will encourage researchers, clinicians, and patients alike to explore CGM data.

Previous open-source resources have been implemented in Excel [8] and R [9], [10] (Table 1). There is currently no comprehensive resource for CGM data in Python, the third most common programming language used globally and the leading language among newcomers to programming [11]. Additionally, previous implementations of open source CGM data analysis present limited metrics of glucose variability. Further, these methods are typically developed for a research specific purpose and without sufficient documentation to be extended to other purposes.

**TABLE 1.** Comparison to Other Open-Source CGM Software

CGM Software	Number of metrics that are clinically validated	Functions for plots?	Python	R	Access
cgmquantify	28	Yes	Yes	Yes	PyPi CRANDBDP MD2K
EasyGV [8]	12	No	No	No	Need to request access
cgmanalysis [9]	21	Yes	No	Yes	CRAN
CGManalyzer [10]	7	Yes	No	Yes	CRAN

The primary goal of this work is to provide a comprehensive, open-source software resource available across both the Python and R programming languages for *systematic and reproducible* analysis of continuous glucose monitor data.

## II. Materials and Methods

cgmquantify is an open-source software Python package and R package composed of 25 functions with 28 clinically validated metrics of glucose and glucose variability, as shown in Table 2. These packages are published under the MIT license.

**TABLE 2.** Glucose and Glucose Variability Metrics

Metric	Description	Equation
interdaySD [6], [7]	Interday standard deviation of glucose	${\sigma _{interday}} = \ \sqrt {\frac{{\sum {{( {{G_{i\ }} - \ \mu } )}^2}}}{N}} $
		Where N = total days, G= glucose value
interdayCV [7]	Interday coefficient of variation of glucose	$C{V_{interday}} = \ \frac{{{\sigma _{interday}}}}{\mu }$
intradaySD Mean [7]	Intraday standard deviation of glucose (mean across all days)	${\sigma _{intrada{y_{mean}} = \ \mathop \sum \limits_N {\sigma _i}}}$
intradayCV Mean [7]	Intraday coefficient of variation of glucose (mean across all days)	$C{V_{intrada{y_{mean}} = \ \mathop \sum \limits_N C{V_i}}}$
intradaySD Median [15]	Intraday standard deviation of glucose (median across all days)	${\sigma _{intrada{y_{median}} = \ median\ ( {{\sigma _i}} )}}$
intradayCV Median [15]	Intraday coefficient of variation of glucose (median across all days)	$C{V_{intrada{y_{median}} = \ median\ ( {C{V_i}} )}}$
intradaySD Standard Deviation [15]	Intraday standard deviation of glucose (standard deviation across all days)	${\sigma _{intrada{y_{standard\ deviation}} = \ SD\ ( {{\sigma _i}} )}}$
intradayCV Standard Deviation [15]	Intraday coefficient of variation of glucose (standard deviation across all days)	$C{V_{intrada{y_{standard\ deviation}} = \ SD\ ( {C{V_i}} )}}$
CONGA24 [6], [16]	Continuous overall net glycemic action over 24 hours	$CONGA24 = \ SD\ (| {{G_t} - \ {G_{t - 24hours}}} |$
GMI [10]	Glucose management indicator	$GMI = \ 3.31 + ( {0.02392*\ \mu \ ( {mg/dL} )} )$
HBGI [6], [7]	High Blood Glucose Index	$\sum \frac{{{r_l}}}{n}$
		${r_{l\ = }}\ 22.7*{( {{\rm{f}}( {{{\rm{G}}_i}} )} )^2}\ if\ f( {{G_{i\ }}} ) \leq 0,\ {r_{l\ = }}0\ otherwise\ $
		$\ f( {{G_i}} ) = \ {\rm{ln}}{({G_i})^{1.084}} + \ 5.381$
LBGI [6], [7]	Low Blood Glucose Index	$\sum \frac{{{r_h}}}{n}$
		${r_{h\ = }}\ 22.7*{( {{\rm{f}}( {{{\rm{G}}_i}} )} )^2}\ if\ f( {{G_{i\ }}} ) > 0,\ {r_{l\ = }}0\ otherwise$
		$f( {{G_i}} ) = \ {\rm{ln}}{({G_i})^{1.084}} + \ 5.381$
ADRR [7]	Average Daily Risk Range, assessment of total daily glucose variations within risk space	$ADRR = \ \frac{{\mathop \sum \nolimits_{all\ days} (L{R^j} + \ H{R^j})}}{{N\ days}}$
		$where\ L{R^j} = \max ( {{r_l}} )and\ H{R^j} = {\rm{max}}( {{r_h}} )$
J-index [6], [17]	Measure of both the mean level and variability of glycemia	$J = 0.001*\ {( {\mu + \ \sigma } )^2}$
MAGE [6], [18]	Mean amplitude of glucose excursions (default = 1SD)	1. Local minima/maxima determined 2. Assess max/min pairs against SD 3. If difference from min to max > SD, mean is retained 4. Otherwise excluded
		Troughs are retained and summed
MGE [6]	Mean of glucose outside range (default = 1SD)	${\mu _{glucose\ outside\ \# \ SD\ of\ mean}}$
		Where # is set, default is 1 SD
MGN*	Mean of glucose inside range (default = 1SD)	${\mu _{glucose\ inside\ \# \ SD\ of\ mean}}$
MODD [7], [16]	Mean of daily differences in glucose	$MODD = \ \frac{{\sum | {{G_t} - \ {G_{t - 24hours}}} |}}{{total\ matched\ observations}}$
TIR [19]	Time spent in range (minutes), default = 1SD	$TIR = \mathop \sum \limits_N time\ inside\ \# \ SD\ of\ mean$
TOR [19]	Time spent outside range (minutes), default = 1SD	$TOR = \ \mathop \sum \limits_N time\ outside\ \# \ SD\ of\ mean$
POR [9]	Percent of time spent outside range	$POR = \ \frac{{TOR}}{{total\ time}}\ x\ 100\% $
PIR [9]	Percent of time spent inside range, default = 1SD	$PIR = \ \frac{{TIR}}{{total\ time}}\ x\ 100\% $
eA1c [20]	Estimated A1c (according to American Diabetes Association)	$eA1c = \ \frac{{( {46.7 + \ \mu \ } )}}{{28.7}}$
meanG [16]	Mean glucose over all days	$\mu = \ \frac{{\mathop \sum \nolimits_N \overline {{x_i}} }}{N}$
medianG [9], [10]	Median glucose over all days	${\rm{median}}( {Glucose} )$
minG [9]	Minimum glucose over all days	${\rm{min}}( {Glucose} )$
maxG [9]	Maximum glucose over all days	${\rm{max}}( {Glucose} )$
Q1G [9]	First quartile glucose value over all days	${\rm{first\ quartile}}( {Glucose} )\ $
Q3G [9]	Third quartile glucose value over all days	${\rm{third\ quartile}}( {Glucose} )$

*indicates previously unidentified metrics of glucose variability that are similar to clinically validated metrics

**FIGURE 1.**
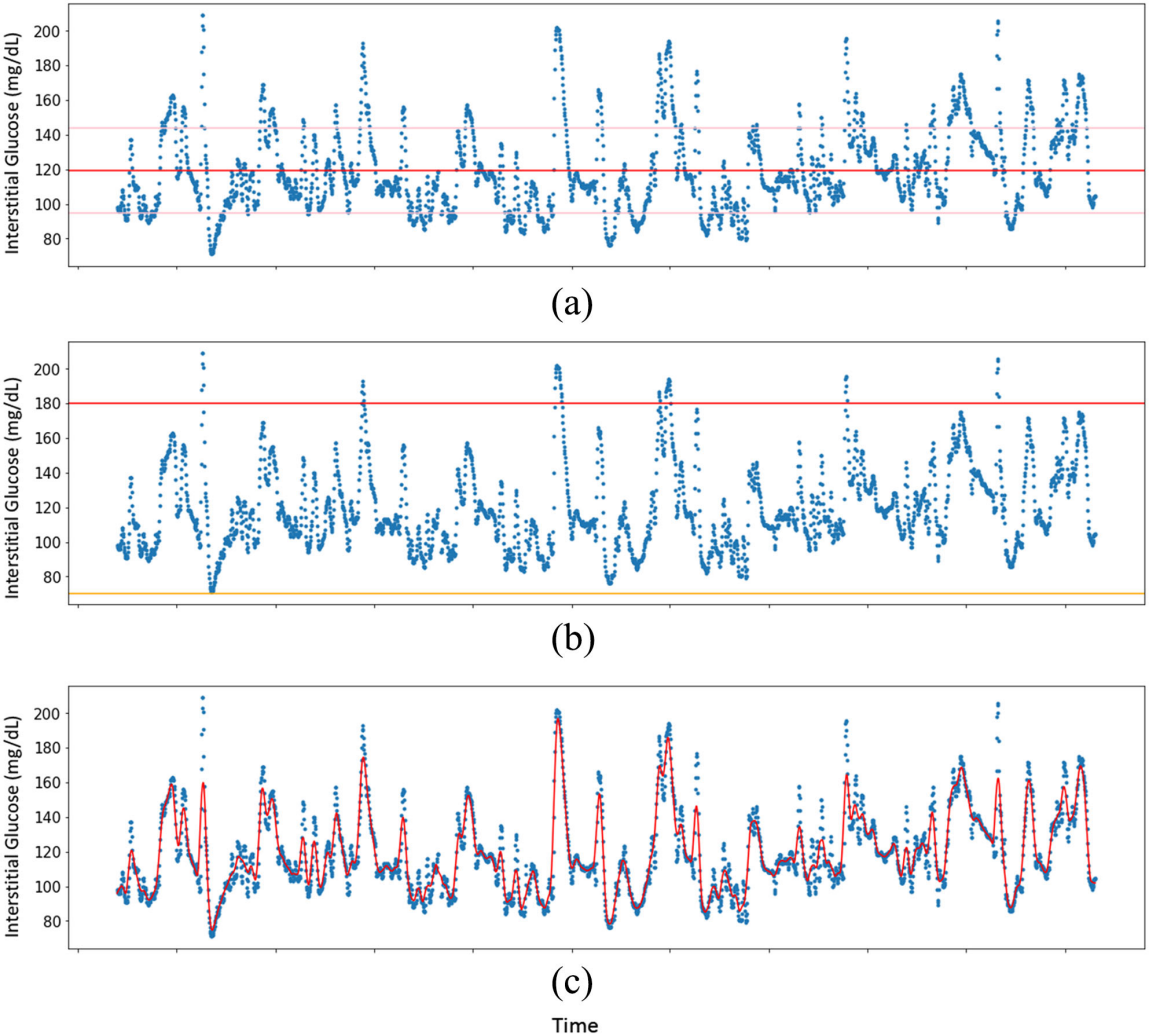
Visualizing longitudinal CGM data with the cgmquantify Python package. Shown are a visualization with indicators of the mean interstitial glucose level (red) and 1 SD from the mean (pink) (a), a visualization with indicators of hyperglycemic (>180 mg/dL glucose, red) and hypoglycemic (<70 mg/dL glucose, yellow) (b), and a plot with LOWESS smoothing of the glucose data (c).

**FIGURE 2.**
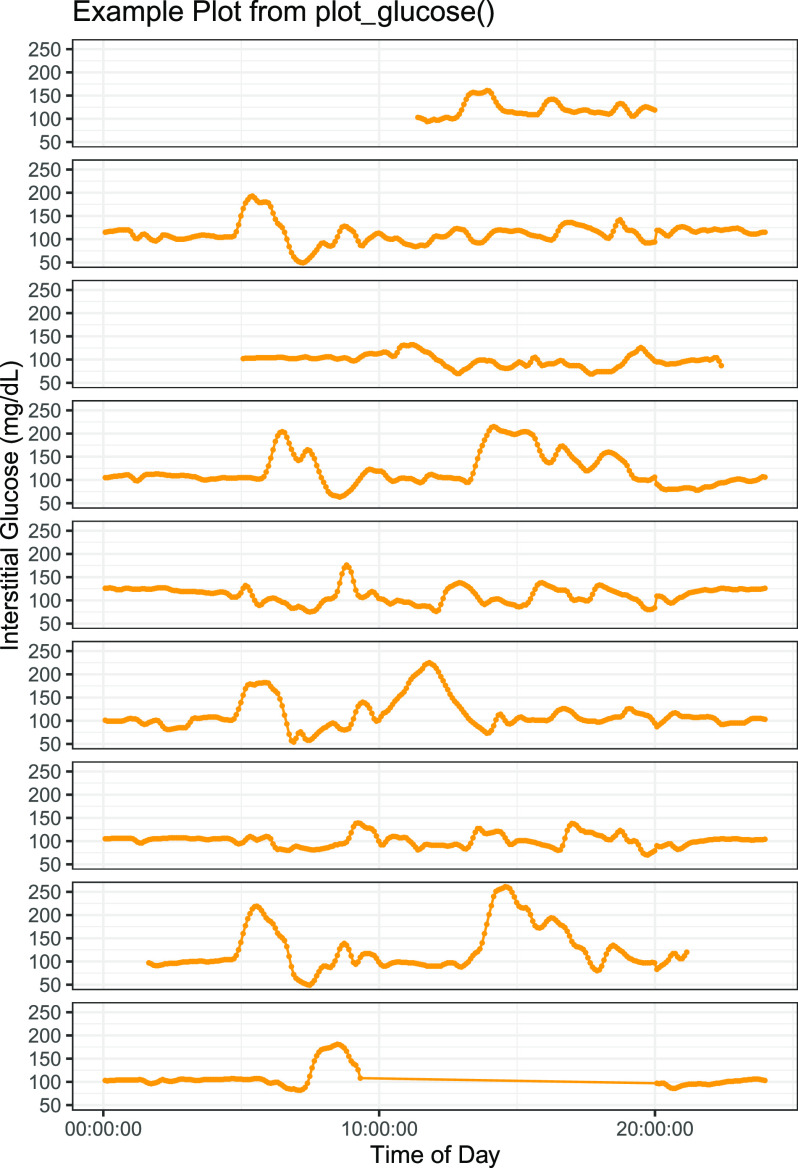
Example plot from the plot_glucose() function from the cgmquantify R package to visualize longitudinal CGM data. Shown is a visualization available in the cgmquantify R package that enables visualization of CGM data by time of day for each day specified.

Customizable visualizations (Fig. 1, Fig. 2) are also included as easy to implement functions.

cgmquantify is version controlled through GitHub, the Python Package Index (PyPI), and the Comprehensive R Archive Network (CRAN). This allows for single-line installation in either language. Source code, an extensive user guide, and issue tracking are available on GitHub to facilitate ease of use and enable customization based on user needs. Tests are available in GitHub under the tests subdirectory to allow for manual testing of all functions. Results of tests of the current software versions are available in GitHub. The current software passes all tests. The community can expand and contribute code to cgmquantify through the DBDP (cite).

The cgmquantify python package is available in PyPi (https://pypi.org/project/cgmquantify/). Source code can be found at https://github.com/DigitalBiomarkerDiscovery Pipeline/cgmquantify. The cgmquantify R package is available in CRAN (https://CRAN.R-project.org/package=cgmquantify) and source code can be found at https://github.com/DigitalBiomarkerDiscoveryPipeline/cgmquantify-R.

## III. Results

We have included import functions to format data for use with the cgmquantify package. These functions currently support Dexcom and Abbott Freestyle Libre CGM devices, with ongoing work adding data import functions for other CGM manufacturers (e.g., Medtronic). Our user guide also outlines how new data can be easily formatted data to make it compatible with the functions in cgmquantify.

Functions are available for all of the commonly studied glucose and glucose variability metrics (Table 2). Additionally, functions for data visualization of the longitudinal CGM data are provided. These visualizations are easily customizable and can include personalized or standard clinical thresholds for hyper- and hypoglycemia. We have also implemented a function that enables LOWESS smoothing over the CGM data to facilitate viewing and interpretation (Fig. 1).

We have integrated cgmquantify in the Digital Biomarker Discovery Pipeline, an open-source software resource aimed at making digital biomarker development and digital medicine more accessible [12]. cgmquantify has also been integrated in MD2K Cerebral Cortex in the MD2K Center of Excellence for turning mobile sensor data into reliable and actionable health information [13]. The cgmquantify software package is already being used in several research projects at multiple institutions.

## IV. Discussion

cgmquantify is a package that simplifies the process of calculating interstitial glucose metrics from CGM data and thus allows for easy comparison across different research studies that use different metrics summarizing glucose and glucose variability. Functions have been developed using equations from clinically validated research studies such that users can compare their results to previous findings. The cgmquantify package is easily implemented with a one-line installation and contains an extensive user guide in both the Python and R languages. Detailed documentation facilitates modification of existing code for customization of input and visualizations. With the aim of build a community of developers to contribute to the literature in this burgeoning field, we anticipate that many researchers will not only find this resource useful for their own work but will contribute code updates and improvements through the DBDP to ensure that this package remains both relevant and useful for the community.

This is a much-needed resource for the community of researchers, clinicians, and patients using CGM. Currently, little is understood about the relationships between glucose and glucose variability metrics from CGM data to diseases including but not limited to prediabetes, Type 2 diabetes, and severity of symptoms in T1D. As more researchers and clinicians start looking to CGM data to answer these questions, the need for a standardized resource available in the most common programming languages is necessary. As we have seen with the Open APS community, analysis of CGM data is not limited to researchers and clinicians but includes patients as well [14]. By providing this toolbox as an open-source resource, we hope to encourage patients to interact with their own data, determine personalized insights, and make meaningful contributions to the digital health landscape.

While the primary use case for cgmquantify is in research, in future directions we may incorporate a graphical user interface to enable ease of use outside of the Python or R programming languages.

## V. Conclusion

The cgmquantify software resource enables comprehensive analysis of continuous glucose monitor data with clinically validated metrics and easy to implement visualizations. Future implementations include incorporating food diary and physical activity data into the visualizations. The fields of digital biomarker development and diabetes research will benefit greatly from this resource.
